# Neonatal Screening for SCID: The French Experience

**DOI:** 10.3390/ijns7030042

**Published:** 2021-07-12

**Authors:** Marie Audrain, Caroline Thomas

**Affiliations:** 1 Laboratoire d’Immunologie, CHU Nantes, 9 Quai Moncousu, CEDEX 1, 44093 Nantes, France; 2 Service d’Oncologie-Hématologie et Immunologie Pédiatrique, 5e Etage, Hôpital Enfant–Adolescent, CHU Nantes, Quai Moncousu, CEDEX 1, 44093 Nantes, France; caroline.thomas@chu-nantes.fr

**Keywords:** SCID, neonatal screening, TREC quantification

## Abstract

After it was demonstrated in 2005 that T cell receptor excision circle (TREC) quantification for dried blood spot (DBS) samples on Guthrie cards is an effective means of SCID screening and following several pilot studies, the practice was formally recommended in the US in 2010. More and more countries have adopted it since then. In France, before the health authorities could recommend adding SCID to the list of five diseases that were routinely screened for, feasibility and cost-effectiveness studies had to be conducted with a sufficiently large cohort of neonates. We carried out three such studies: The first sought to verify the effectiveness of the assay. The second, DEPISTREC, evaluated the feasibility of universal SCID screening in France and assessed the clinical benefit and economic advantage it would provide. Through the third study, NeoSKID, still under way and to continue until recommendations are issued, we have been offering SCID screening in the Pays de la Loire region of France. This review briefly describes routine newborn screening (NBS) and management of primary immunodeficiency diseases (PIDs) in France, and then considers the lessons from our studies and the status of SCID screening implementation within the country.

## 1. Introduction

In 2005, Chan and Puck [1] demonstrated that T cell receptor excision circles (TRECs) are undetectable in peripheral blood from SCID neonates. They also showed that TRECs could be quantified using dried blood spot (DBS) samples from Guthrie cards after DNA extraction and TREC amplification. Pilot studies were then conducted in Wisconsin and Massachusetts [2,3], and proved the feasibility and clinical utility of TREC quantification. After the Secretary of the US Department of Health and Human Services’ Advisory Committee on Heritable Disorders in Newborns and Children recommended newborn screening (NBS) for SCID through TREC quantification in 2010 [4], several US states implemented it and other countries [5] followed suit, arguing that it was cost-effective: early detection means early treatment with hematopoietic stem cells (HSCs), before any infection occurs, and thus greater survival [6,7].

Currently, France screens 753,000 babies annually (2020 data) through DBS NBS, testing for six diseases: phenylketonuria (since 1972); congenital hypothyroidism (1978); congenital adrenal hyperplasia (1995); sickle-cell anemia (1995), for a subset of the population more likely to develop the disease; cystic fibrosis (2002); and medium-chain acyl-CoA dehydrogenase deficiency (2020, though first recommended by the French National Authority for Health (HAS) in 2011), using mass spectrometry. DBS samples are usually collected on day 3. In addition to DBS NBS, hearing tests on day 2 have been routine since 2014.

**Figure 1:**
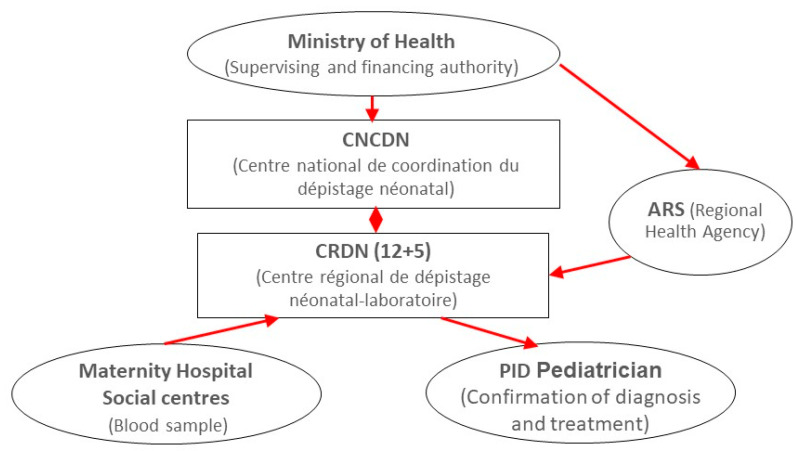
The French NBS program.

The French Ministry of Health supervises the national NBS program and funds it through the regional health agencies (ARSs) (Figure 1). A national center (CNCDN) acts as coordinating body [8]. Seventeen regional screening centers (CRDNs)—12 in mainland France and 5 overseas, each associated with a university hospital and a regional health agency—collect DBSs from the maternity hospitals and perform the tests. Each center has identified pediatricians specialized in primary immunodeficiency diseases (PIDs), who confirm NBS diagnoses and implement treatment programs. Decisions to add a new disease to the NBS battery are taken by the Ministry of Health after considering HAS recommendations formulated on the basis of data from clinical trials and the literature.

Under the French national rare disease plan (PNMR), the study, diagnosis, and treatment of rare diseases such as SCID are conducted through dedicated networks, each consisting of a single national center of expertise (CRMR) relayed by regional centers of expertise (CCMRs) and laboratories, thus ensuring equal access to care. The national center of expertise for SCID and other PIDs is the CEREDIH (Center of Expertise in Primary Immune Deficiencies), located at Necker Hospital in Paris [9]. Diagnoses are made by pediatricians or internists specialized in PIDs. For SCID in particular, molecular diagnosis and treatment are usually provided through the CEREDIH, but occasionally through regional centers.

Before the authorities could recommend including SCID, feasibility and cost-effectiveness studies with a large cohort of newborns were required. As several screening methods had been published, we conducted a preliminary single-center study in 2012. It was not a pilot study: rather, it sought to demonstrate that the selected method of TREC quantification—the TREC assay described by the Massachusetts-based team of Dr Comeau [10]—met our criteria of specificity, reproducibility, and affordability. Briefly, between June and October 2012, 5028 unselected, de-identified newborn DBS samples and 8 DBS samples from SCID patients were collected. TRECs were detected for all 5028 unselected samples, and results were equivocal for 2. As samples were de-identified, no other DBSs could be requested for verification nor any referrals made. Of the 8 SCID patients, 7 had no detectable TRECs, and results were equivocal for the remaining child (ZAP70 deficiency). The method was effective and yielded a recall rate of only 0.04% at the chosen cutoff [11].

In 2015, a preliminary French study conducted by the URC—Eco Île de France health economics research unit—and the CEREDIH compared children diagnosed without screening and receiving HSC treatment after the age of 3 months with others diagnosed early, through knowledge of family history, and treated before the age of 3 months [12]. The study concluded that universal NBS for SCID is probably cost-effective if early treatment improves survival. The cost of screening should be offset by savings elsewhere.

We were ready to proceed to the next step, a large multicenter cost-effectiveness study to assess the feasibility of universal SCID screening in France, and to demonstrate the clinical benefit and economic advantage it would offer. To fulfill its purpose, this study would need to include 200,000 children from across France.

Accordingly, the DEPISTREC study was conducted between January 2015 and March 2017, with a grant from the French Ministry of Health. Its results were published in 2018 and 2019 [13,14]. Unfortunately, official recommendations on SCID NBS have yet to be issued. In October 2019, after a lengthy pause, we began to offer routine SCID screening in the Pays de la Loire region of France—for all babies born there—through the NeoSKID program. The aim of this program, which we intend to pursue until national recommendations are released, is to continue analyzing clinical data and identify any other causes that contribute to abnormal results.

In this review, we will summarize the different steps and pitfalls encountered in the implementation of SCID NBS in France, as presented at the last meeting of the International Society for Neonatal Screening and UK NBS network in January 2021.

## 2. Overview of the DEPISTREC Pilot Study

In 2013, Nantes University Hospital (CHU de Nantes) received a grant (PRME-13-0265) from the French Ministry of Health to conduct a health economics research program (ClinicalTrials.gov Identifier: NCT02244450). In all, 2 laboratories and 48 maternity wards from across France were to participate in the nationwide multicenter prospective study, which was conducted in parallel with routine screening from January 2015 to March 2017 [8,9].

The aim of the two-year DEPISTREC study was to assess the feasibility, clinical utility, and cost-effectiveness of routine SCID NBS in France. For this purpose, it needed to include about 200,000 newborns—in order to have a 95% chance of at least one SCID case (Poisson distribution) at an expected prevalence of 1/70,000. The screened infants in the experimental group were compared with a control group of unscreened children diagnosed with SCID during the same period. The study protocol was approved by the ethical review board of Nantes.

DBS samples—in addition to the routine DBS samples—were collected from 3-day-old infants on a single separate Guthrie card, at the 48 participating maternity wards. Samples were sent twice a week to the 2 labs for analysis with the CE-marked EnLite Neonatal TREC in vitro diagnostic kit (PerkinElmer).

**Figure 2:**
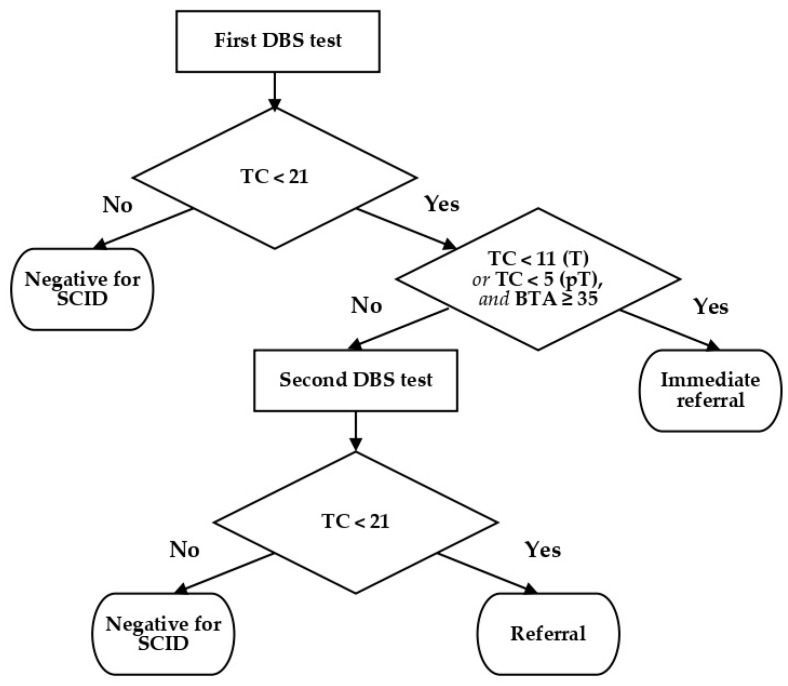
Decision-making algorithm. BTA = beta-actin count (copies/µL); DBS = dried blood spot; T = term; pT = preterm; TC = TREC count (copies/µL).

Different algorithms have been described by professionals conducting SCID screening, depending on what is being quantified (i.e., TRECs with or without KRECs) [15]. Some propose different algorithms for samples from preterm or neonatal ICU infants [16,17]. Most make a distinction between undetectable TREC levels (calling for immediate referral) and low TREC levels (calling for a second DBS sample) [15,18]. At the beginning of the DEPISTREC study, we decided to use a cutoff of 21 TREC copies per microliter. If the initial result was <35 (TREC copies/µL), analyses of two additional punches from the same DBS card were performed. When TREC counts for 2 out of the 3 punches were <21, the test was deemed positive and the infant was referred to a pediatrician specialized in PIDs. An exception was made for preterm babies: for this group, a result of <6 led to immediate referral, while TREC levels between 6 and 21 prompted the request of another DBS. After analyzing the results for 100,000 babies screened using this algorithm, we judged the recall rate to be too high. Because all the SCID babies tested had very low TREC counts (<11), we altered the algorithm to distinguish results of <11 from those between 11 and 21: thenceforth, initial TREC counts of <11 (at term)—or <5 for preterm infants—led to immediate referrals, as long as beta-actin (BTA) levels were >35 copies/µL. Second tests were only requested in other cases where TREC counts were <21 (Figure 2).

We included 190,517 newborns in the screening group and 28 in the control group. The updated algorithm—described in Figure 2 and the preceding paragraph and applied during the second part of the study—concerned the last 72,411 samples. For this stage of the study, after the first DBS test, 0.16% of the infants were recalled for a second DBS, and 0.023% of the babies were recalled for a medical appointment. After testing the second DBS, 0.017% were still positive. Thus, in all, 0.04% of the babies were recalled for appointments and complementary examinations. Assuming 750,000 births annually in France, this would mean approximately 300 babies per year. Furthermore, by applying these criteria, the recall rate (0.04%) was similar to that observed in previous studies, summarized in the review by van der Spek et al. [19]. This recall rate is lower than that observed by Blom et al. in the Netherlands (1295 samples with a cutoff of 22 copies/µL) [17] and higher than that determined by Rechavi in Israel (0.017% of 290,864 samples, using a cutoff of 23 copies/µL) [20], but this discrepancy is explained by differences in procedures.

**Figure 3:**
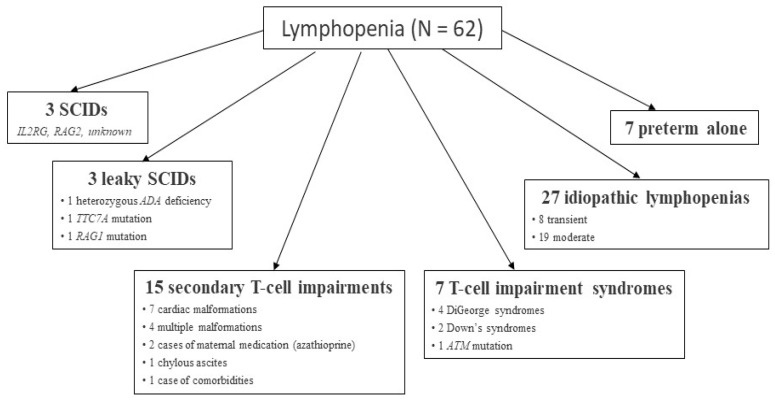
Description of 62 cases of lymphopenia.

For the whole group of 190,517 infants tested, 291 children (0.015%) needed a second DBS, and 165 children were recalled for flow cytometry testing and an appointment with a pediatrician specialized in PIDs. Of these 165 children, flow cytometry analyses were not performed for 25, either because they had died or because we received no further information about them. Among the remaining 140 infants recalled, 62 suffered from lymphopenia [14] due to SCID (3 cases) or secondary causes (Figure 3), as has previously been described [21,22].

In one of the two cases of maternal medication with azathioprine, the infant had a homozygous *TPMT* mutation, undetectable TRECs, and <300 CD3^+^ lymphocytes per microliter, with levels returning to normal in 3 months [23]. This case has previously been discussed in the literature [21,22].

Thus, the incidence of SCID in this study was 1 out of 63,500 live births, which is comparable to what has been reported in the literature since (and as a result of) the advent of SCID NBS [21]. Our results may be interpreted as indicating 162 false positives for SCID and 103 false positives for lymphopenia. This yields a PPV of 1.8% for SCID. By comparison, published rates range from 0.8% to 20% [19]. Though this is very low, one must remember that screening, not diagnosis, is in question. Still, the higher the rate, the lower the anxiety for parents of infants recalled for further testing or an appointment. We could not calculate the NPV because false negatives were impossible to identify.

In the control group (*n* = 28), two were diagnosed with SCID through knowledge of their family history. Retrospective TREC screening was performed on neonatal DBS samples from 21 of these children: in all cases, screening results were positive, suggesting 100% sensitivity and that SCID NBS might have diagnosed their conditions at birth. Furthermore, 5 children from this group died with severe infections due to SCID before they could receive HSC transplants. It is very likely that diagnosis through neonatal TREC analysis could have prevented these deaths.

Health economics analysis of DEPISTREC data made it possible to calculate the cost of testing. However, it did not permit confirmation of the benefit, in terms of average cost, that earlier HSC transplantation prompted by positive screening results would have, due to the very small number of children in the experimental group diagnosed with SCID (3 babies, 1 of whom died). The test is relatively cheap. Its cost was estimated at $4.22 in the US, by Chan et al. [24], and €4.21 by the DEPISTREC study (covering reagents, instrumentation, and staff). Given the rareness of SCID and the cost of its treatment, health economics studies are needed to encourage adoption of routine SCID screening by countries’ health authorities.

## 3. Initial Findings of the NeoSKID Program

Since October 2019, through the ongoing NeoSKID program approved by the ethical review board of Nantes, we have continued to offer SCID NBS through TREC quantification at the Pays de la Loire regional screening center for all children born in the region. Between October 2019 and April 2021, 58,645 samples were tested.

For this set of samples, the percentage of babies recalled for a second DBS was 0.22%, while 0.02% were recalled for medical appointments. T cell lymphopenia (<1500 CD3^+^ lymphocytes/µL) was identified in 3 children (0.005%, or 1/20,000). Among them, 1 ADA deficiency was identified in a girl who is currently well into treatment with ADA and waiting for an HSC graft. One other newborn, with undetectable TRECs, had an antenatal diagnosis and family history of JAK3 deficiency, though we were unaware of this at the time of screening. The third abnormal result was from a newborn with Down syndrome. For the 58,645 samples tested, results were available 10 to 12 days after birth if normal, or 13 days after birth if abnormal. This turnaround reflects mail delivery time and the fact that SCID screening is currently performed after NBS for other diseases. Table 1 shows a comparison of the recall rates for the second stage of the DEPISTREC study and the NeoSKID program, using the same algorithm.

## 4. DEPISTREC and NeoSKID: Lessons Learned

### 4.1. Feasibility and Clinical Utility

We showed that screening for SCID is both feasible and effective and offers the added benefit of detecting severe T cell lymphopenia in children with non-SCID disorders such as DiGeorge syndrome, Down syndrome, ataxia-telangiectasia, and congenital heart diseases or syndromes, or in children who have been affected by maternal immunosuppressive treatment. Thus, these children may likewise benefit from treatment upon detection of their lymphopenia. SCID screening can be performed using the same DBS samples currently collected: an additional Guthrie card need not be prepared. In our experience, not more than 0.04% of the babies were recalled for appointments and flow cytometry analysis. Finally, the turnaround for results is <15 days.

### 4.2. Reagents and Cut Off Values

We encountered some difficulties concerning the standardization of reagents. During the study, several kit lots were used, and TREC count medians and percentiles differed between them. The 0.5th percentile, for example, ranged from 19 to 26, highlighting the challenge of establishing cutoff values. Curiously and fortunately, this did not change the percentage of positive samples (Table 2).

In all, 151 DBS samples found to be abnormal by one laboratory were sent to the other one for retesting: in 25% of the cases, the other laboratory obtained contradictory results. This always concerned values near the cutoff, and results were normal for the second DBS test in all but one case (post-thymectomy).

Nevertheless, while the percentages of babies recalled for a new DBS differed between the two laboratories, the percentages of babies recalled for appointments were nearly identical (Table 3). The larger number of DBS recalls for Lab 2 might reflect the higher percentage of preterm babies (Lab 1: 8.5%; Lab 2: 9.5%), which is probably related to the population served by the participating centers.

If SCID NBS is carried out at 12 different regional centers, this variability could still be a problem. Thus, for reasons both technical and economic (the unit cost of diagnosis varied with the volume of laboratory work), we recommend a small number of testing laboratories. The cutoff value is also important and depends on what we want to detect: SCID only or other severe T cell lymphopenias as well? The choice of cutoff is always a balance between sensitivity and specificity, and it must be kept in mind that recalls for second DBS samples or appointments can generate anxiety in children’s families. Based on DEPISTREC data and our testing of SCID babies before that study, SCID-positive TREC counts are always <11 copies per microliter. Still, a late ADA deficiency may be associated with slightly higher TREC counts, yielding an equivocal or even a normal result.

### 4.3. Preterm Babies

We also showed through the DEPISTREC study, as previously described, that TREC counts could be lower for preterm infants, causing higher rates of false positives, and that there was a correlation between TREC counts and prematurity. Although the overall recall rate (i.e., for either a second DBS sample or a doctor’s appointment) was significantly higher (*p* < 0.0001) for preterm (1.36%) than for term (0.12%) babies, we do not believe that the cutoff value needs to be changed for preterm babies. Overall and second-DBS recall rates for each gestational-age subgroup of premature infants were always significantly higher than for the next older subgroup [25]—that is, the younger the gestational age, the higher the recall rate. In view of the literature and our findings, we initially suggested the following protocol for the screening of preterm infants: DBS samples should be collected during the first days of life (usually at around 72 h), regardless of infants’ gestational age, so that they may be quickly treated if no TRECs are detected. If the initial DBS sample is positive but TREC levels exceed the chosen cutoff value, a second sample should be collected near term (around 37 weeks of gestational age). This delay minimizes the chance of a false positive but is sufficiently brief to avoid losing track of the child, since preterm babies may still be hospitalized at that point. If results are abnormal for the second DBS sample, a lymphocyte subset analysis needs to be carried out and an appointment with a primary care pediatrician scheduled.

However, since October 2019, we have observed that obtaining a second DBS at 37 weeks of gestational age can be very difficult. Accordingly, the delay we had initially suggested might not be advisable. Though other strategies are a possibility, we have for now settled on requesting another DBS as soon as we obtain abnormal results for the first, to avoid losing track of infants. The second DBS usually tests normal.

### 4.4. Conclusions

Test validation rules must be clearly defined and the decision-making algorithm leading up to diagnosis and treatment standardized, so that every child has the same chances regardless of where they are born. When SCID NBS results are positive, the lymphopenia detected may not be SCID and must be diagnosed, just as is done for a child identified without screening. The CEREDIH ensures that these procedures are firmly established at the regional centers within its nationwide network.

## 5. Where Are We Now?

Adding a new disease to the French national NBS program is a long road. The decision must be made by the Ministry of Health, on the basis of HAS recommendations—informed by the literature or, ideally, data from pilot studies. Since candidate and current NBS diseases are rare, these pilot studies must include a large number of babies to be valid, are expensive, and can be very long. Thus, it took 2 years for us to receive the grant for the DEPISTREC study, 1 year to obtain all authorizations and prepare, and 3½ years to conduct the study and publish findings. In 2017, the Ministry of Health asked the HAS to issue recommendations on SCID NBS, which we have been awaiting since the end of the DEPISTREC study in April 2018. They were expected initially for the third quarter of 2019 and then for the first quarter of 2021 [26,27]. We hope that SCID NBS will be highly recommended so that affected children can be diagnosed and treated at a presymptomatic stage, before any infection, thereby saving lives. The DEPISTREC and NeoSKID studies have demonstrated the feasibility of screening in France, and monitoring the SCID screening process there should confirm its health economics value.

## Figures and Tables

**Table 1:** Recall rates for SCID screening.

	DEPISTREC Study	NeoSKID Program
Number of samples	72,411	58,645
Recall rate for a 2nd DBS	0.16%	0.22%
Recall rate for appointments	0.023%	0.026%
Recall rate for appointment after 2nd DBS	0.017%	0.005%
Total recall rate for appointment and flow cytometry	0.04%	0.03%

DBS = dried blood spot.

**Table 2:** Kit lot variation.

Kit Lot Code	1	2	3	4	5	6
Median TREC count	110	153	88	118	111	122
0.5th percentile	21	25	19	25	24	26
Positive samples (%)	0.12	0.12	0.14	0.11	0.13	0.12

**Table 3:** Recall rates for each laboratory.

	Lab 1	Lab 2
*N*	49,145	23,266
Abnormal results, %	0.15	0.25
Second DBS test, %	0.13	0.22
Immediate referral, %	0.022	0.026
Referral—immediate or after the second DBS, %	0.039	0.043

## References

[1] Chan K., Puck J.M. (2005). Development of Population-Based Newborn Screening for Severe Combined Immunodeficiency. J. Allergy Clin. Immunol..

[2] Routes J.M., Grossman W.J., Verbsky J., Laessig R.H., Hoffman G.L., Brokopp C.D., Baker M.W. (2009). Statewide Newborn Screening for Severe T-Cell Lymphopenia. JAMA.

[3] Comeau A.M., Hale J.E., Pai S.-Y., Bonilla F.A., Notarangelo L.D., Pasternack M.S., Meissner H.C., Cooper E.R., DeMaria A., Sahai I. (2010). Guidelines for Implementation of Population-Based Newborn Screening for Severe Combined Immunodeficiency. J. Inherit. Metab. Dis..

[4] Advisory Committee on Heritable Disorders in Newborns and Children, Summary of 20th Meeting, Washington, DC, USA, 21–22 January 2010. https://www.hrsa.gov/sites/default/files/hrsa/advisory-committees/heritable-disorders/meetings/Heritable%20Disorders%202004-2015/2010/January%2021-22,%202010/minutes.pdf.

[5] PID Life Index: Map. https://pidlifeindex.ipopi.org/#/en/principles/world-map.

[6] Buckley R.H., Schiff S.E., Schiff R.I., Markert M.L., Williams L.W., Roberts J.L., Myers L.A., Ward F.E. (1998). Hematopoietic stem-cell transplantation for the treatment of severe combined immunodeficiency. N. Engl. J. Med..

[7] Pai S.-Y., Logan B.R., Griffith L.M., Buckley R.H., Parrott R.E., Dvorak C.C., Kapoor N., Hanson I.C., Filipovich A.H., Jyonouchi S. (2014). Transplantation outcomes for severe combined immunodeficiency, 2000–2009. N. Engl. J. Med..

[8] Ministère des Solidarités et de la Santé [French Ministry of Solidarity and Health] Programme National de Dépistage Néonatal [French National Newborn Screening Program]. https://solidarites-sante.gouv.fr/soins-et-maladies/prises-en-charge-specialisees/maladies-rares/DNN.

[9] CEREDIH. https://www.ceredih.fr/.

[10] Gerstel-Thompson J.L., Wilkey J.F., Baptiste J.C., Navas J.S., Pai S.-Y., Pass K.A., Eaton R.B., Comeau A.M. (2010). High-Throughput Multiplexed T-Cell-Receptor Excision Circle Quantitative PCR Assay with Internal Controls for Detection of Severe Combined Immunodeficiency in Population-Based Newborn Screening. Clin. Chem..

[11] Audrain M., Thomas C., Mirallie S., Bourgeois N., Sebille V., Rabetrano H., Durand-Zaleski I., Boisson R., Persyn M., Pierres C. (2014). Evaluation of the T-Cell Receptor Excision Circle Assay Performances for Severe Combined Immunodeficiency Neonatal Screening on Guthrie Cards in a French Single Centre Study. Clin. Immunol..

[12] Clément M.C., Mahlaoui N., Mignot C., Le Bihan C., Rabetrano H., Hoang L., Neven B., Moshous D., Cavazzana M., Blanche S. (2015). Systematic Neonatal Screening for Severe Combined Immunodeficiency and Severe T-Cell Lymphopenia: Analysis of Cost-Effectiveness Based on French Real Field Data. J. Allergy Clin. Immunol..

[13] Audrain M.A.P., Léger A.J.C., Hémont C.A.F., Mirallié S.M., Cheillan D., Rimbert M.G.M., Le Thuaut A.M.-P., Sébille-Rivain V.A., Prat A., Pinel E.M.Q. (2018). Newborn Screening for Severe Combined Immunodeficiency: Analytic and Clinical Performance of the T Cell Receptor Excision Circle Assay in France (DEPISTREC Study). J. Clin. Immunol..

[14] Thomas C., Durand-Zaleski I., Frenkiel J., Mirallié S., Léger A., Cheillan D., Picard C., Mahlaoui N., Riche V.-P., Roussey M. (2019). Clinical and Economic Aspects of Newborn Screening for Severe Combined Immunodeficiency: DEPISTREC Study Results. Clin. Immunol..

[15] Trück J., Prader S., Natalucci G., Hagmann C., Brotschi B., Kelly J., Bassler D., Steindl K., Rauch A., Baumgartner M. (2020). Swiss newborn screening for severe T and B cell deficiency with a combined TREC/KREC assay—Management recommendations. Swiss Med. Wkly..

[16] Dorsey M., Puck J. (2017). Newborn Screening for Severe Combined Immunodeficiency in the US: Current Status and Approach to Management. Int. J. Neonatal Screen..

[17] Blom M., Pico-Knijnenburg I., Sijne-van Veen M., Boelen A., Bredius R.G.M., van der Burg M., Schielen P.C.J.I. (2017). An evaluation of the TREC assay with regard to the integration of SCID screening into the Dutch newborn screening program. Clin. Immunol..

[18] Argudo-Ramírez A., Martín-Nalda A., Marín-Soria J.L., López-Galera R.M., Pajares-García S., González de Aledo-Castillo J.M., Martínez-Gallo M., García-Prat M., Colobran R., Riviere J.G. (2019). First Universal Newborn Screening Program for Severe Combined Immunodeficiency in Europe: Two-Years’ Experience in Catalonia (Spain). Front. Immunol..

[19] van der Spek J., Groenwold R.H., van der Burg M., van Montfrans J.M. (2015). TREC Based Newborn Screening for Severe Combined Immunodeficiency Disease: A Systematic Review. J. Clin. Immunol..

[20] Rechavi E., Lev A., Saraf-Levy T., Etzioni A., Almashanu S., Somech R. (2017). Newborn Screening for Severe Combined Immunodeficiency in Israel. Int. J. Neonatal Screen..

[21] Kwan A., Abraham R.S., Currier R., Brower A., Andruszewski K., Abbott J.K., Baker M., Ballow M., Bartoshesky L.E., Bonilla F.A. (2014). Newborn Screening for Severe Combined Immunodeficiency in 11 Screening Programs in the United States. JAMA.

[22] Jyonouchi S., Jongco A.M., Puck J., Sullivan K.E. (2017). Immunodeficiencies Associated with Abnormal Newborn Screening for T Cell and B Cell Lymphopenia. J. Clin. Immunol..

[23] Thomas C., Monteil-Ganiere C., Mirallié S., Hémont C., Dert C., Léger A., Joyau C., Caldari D., Audrain M. (2018). A Severe Neonatal Lymphopenia Associated with Administration of Azathioprine to the Mother in a Context of Crohn’s Disease. J. Crohns Colitis.

[24] Chan K., Davis J., Pai S.-Y., Bonilla F.A., Puck J.M., Apkon M. (2011). A Markov Model to Analyze Cost-Effectiveness of Screening for Severe Combined Immunodeficiency (SCID). Mol. Genet. Metab..

[25] Thomas C., Hubert G., Catteau A., Danielo M., Riche V.P., Mahlaoui N., Moshous D., Audrain M. (2020). Review: Why Screen for Severe Combined Immunodeficiency Disease?. Arch. Pediatr..

[26] Haute Autorité de Santé [French National Authority for Health] (2018). Feuille de Route: Evaluation a Priori de l’Extension du Dépistage Néonatal au Déficit Immunitaire Combiné Sévère (DICS) [Roadmap: Preliminary Evaluation of Extension of Newborn Screening Coverage to SCID]. https://www.has-sante.fr/upload/docs/application/pdf/2018-08/feuille_de_route_evaluation_a_priori_de_lextension_du_depistage_neonatal_au_deficit_immunitaire_combine_severe_dics.pdf.

[27] Haute Autorité de Santé (2020). Programme de Travail. https://www.has-sante.fr/upload/docs/application/pdf/2020-07/programme_de_travail_has_2020.pdf.

